# Identification of functional features underlying heat stress response in Sprague–Dawley rats using mixed linear models

**DOI:** 10.1038/s41598-022-11701-y

**Published:** 2022-05-10

**Authors:** Krzysztof Kotlarz, Magda Mielczarek, Yachun Wang, Jinhuan Dou, Tomasz Suchocki, Joanna Szyda

**Affiliations:** 1 Biostatistics Group, Department of Genetics, Wroclaw University of Environmental and Life Sciences, Kozuchowska 7, 51-631 Wroclaw, Poland; 2 National Research Institute of Animal Production, Krakowska 1, 32-083 Balice, Poland; 3 Key Laboratory of Animal Genetics, Breeding and Reproduction, MARA, National Engineering Laboratory for Animal Breeding, College of Animal Science and Technology, China Agricultural University, Beijing, 100193 China; 4 College of Animal Science and Technology, Beijing University of Agriculture, Beijing, 102206 China

**Keywords:** Gene expression, Systems biology

## Abstract

Since global temperature is expected to rise by 2 °C in 2050 heat stress may become the most severe environmental factor. In the study, we illustrate the application of mixed linear models for the analysis of whole transcriptome expression in livers and adrenal tissues of Sprague–Dawley rats obtained by a heat stress experiment. By applying those models, we considered four sources of variation in transcript expression, comprising transcripts (1), genes (2), Gene Ontology terms (3), and Reactome pathways (4) and focussed on accounting for the similarity within each source, which was expressed as a covariance matrix. Models based on transcripts or genes levels explained a larger proportion of log_2_ fold change than models fitting the functional components of Gene Ontology terms or Reactome pathways. In the liver, among the most significant genes were *PNKD* and *TRIP12*. In the adrenal tissue, one transcript of the *SUCO* gene was expressed more strongly in the control group than in the heat-stress group. *PLEC* had two transcripts, which were significantly overexpressed in the heat-stress group. *PER3* was significant only on gene level. Moving to the functional scale, five Gene Ontologies and one Reactome pathway were significant in the liver. They can be grouped into ontologies related to DNA repair, histone ubiquitination, the regulation of embryonic development and cytoplasmic translation. Linear mixed models are valuable tools for the analysis of high-throughput biological data. Their main advantages are the possibility to incorporate information on covariance between observations and circumventing the problem of multiple testing.

## Introduction

The most general formulation of heat stress is defined as an increase in ambient temperature above the threshold beyond which body temperature cannot be maintained on the physiologically optimal level^1,2^. Heat stress is among the best characterized environmental stressors and has the most severe detrimental effects. Individuals cope with heat stress by increasing body temperature, reducing feed intake as well as changing of physiological state, which is referred to as physiological adaptive responses^3^. Therefore, heat stress can lead to economic losses due to lower productivity and reproductivity, as well as increased health burden^4^. Unfortunately, global temperature is expected to rise by 2 °C in 2050^5^ and furthermore, the average temperature in 2100 may rise by up to 4.8 °C^6^. As a consequence, the maximum daily temperature will be higher, so the intensity and duration of heat stress will be more severe and prolonged, compared to 2021. Therefore, heat stress may become the most severe environmental factor.

Studies have reported that heat stress significantly altered physiological, biochemical, metabolic, and cellular responses in mice and livestock models^3,7,8^. However, animals have developed protective measures against the challenges of heat stress manifested by altered transcript and gene expression levels^9–11^. With the development of next generation sequencing technologies, including RNA-sequencing (RNA-seq), hundreds or thousands of genes involved in heat stress response have been identified^12,13^. Consequently, the main goal of the research was to illustrate how linear mixed models can be used to perform a biologically driven analysis of changes in transcript and gene expression levels between the control and the heat-stress groups. In contrast to the vast majority of approaches, when particular transcripts are considered as independent, we incorporated biological information on the correlation between them directly into the statistical model. Furthermore, we also considered higher-order units, expressed by Gene Ontology terms and Reactome pathways, since these are the actual functional components of the physiological response towards heat stress.

The incorporation of functional units into the analysis of RNA-seq data is a known idea. It has been applied either through enrichment analysis as e.g. by Dou et al.^14^ to the data set analysed in our study or through the Ingenuity Pathway Analysis as e.g. by Lan et al.^15^ to gene expression under heat stress in poultry. Nevertheless, the major difference between our approach and the aforementioned studies is that the former conducts a two-step analysis, by first identifying significantly differentially expressed transcripts and genes by considering them independent (step 1) and then further analysing the selected, significant transcripts and genes in terms of the correlation between them or the enrichment of ontologies or pathways in this significant gene set (step 2). The approach proposed in our study attempted to combine the process within one-step by including the correlation of the functional information directly into the model fitted to the whole (i.e. not only to significant) expression data and is statistically similar to the linear mixed model fitted to gene expression data by Wang et al.^16^, albeit with a different approach to the modelling of the covariance of random effects.

In the current study, we illustrate the application of the mixed models using the whole genome transcript expression data of Sprague–Dawley rats obtained from a heat stress experiment^14^.

## Methods

### Experimental animals

The data set underlying the analysis is a subset of the material used by Dou et al.^14^. In brief, the analysed individuals comprised eight weeks old female, specific-pathogen-free, Sprague–Dawley rats. Prior to the experiment, all rats were housed in a laboratory at 22 ± 1 °C, 50% relative humidity with 12 h reverse light/dark cycle with feed and water provided ad libitum. After one week, five rats from the heat-stressed group were exposed to 42 °C for 120 min, while five rats from the control group were housed at an initial temperature of 22 °C. After the completion of the experiment, the animals were euthanised and samples of liver and adrenal gland tissues were used as a source of total RNA.

### Bioinformatic analysis

Illumina HiSeq2000 was used to sequence 150 long reads in the paired-end (PE) data mode. The total number of read pairs per sample was from 51,706,978 to 97,059,004. The detailed description of the RNA isolation and sequencing was also provided by Dou et al.^14^. The bioinformatic pipeline consisted of the following steps: quality control of raw reads, editing of raw reads based on their quality, and quantification of transcripts’ expression. In particular, the quality of raw reads was assessed by applying the FastQC software^17^. Then, reads were processed by the Trimmomatic software^18^, which removed adapter sequences, trimmed reads with an average sequencing quality of 4 consecutive reads below 20 (*SLIDINGWINDOW:4:20*), and removed reads shorter than 60 bp (*MINLEN:60*). Next, the Salmon software^19^ was used to quantify the abundances of transcripts. This software implements the pseudoalignment process—an approach allowing for rapid identification of the compatibility of reads with transcripts, without the need of a computationally intensive whole genome alignment. In the last step, log_2_ fold changes in transcript expression levels between the control and heat stressed groups were calculated using DESeq2^20^.

### Statistical modelling of expression data

The log_2_ fold changes (log_2_FC) calculated based on the transcript expression levels pooled over the control and heat-stressed animals respectively, were analysed in four mixed linear models.

The *transcript-based model* (M1) is given by:

1 $${\varvec{y}}={\varvec{\mu}}+{{\varvec{Z}}}_{M1}{\varvec{t}}+{{\varvec{e}}}_{M1},$$

where ***y*** is the vector of log_2_FC of transcript expression, ${\varvec{\mu}}$ represents the general mean, ${\varvec{t}}$ is the random transcript effect with a predisposed normal distribution defined by $N\left(0,{{{\varvec{V}}}_{M1}\sigma }_{t}^{2}\right)$, ${{\varvec{e}}}_{M1}$ is a vector of residuals distributed as $N\left(0,{{\varvec{I}}\sigma }_{{e}_{M1}}^{2}\right)$, ${{\varvec{Z}}}_{M1}$ is an incidence matrix for ${\varvec{t}}$. In this model, the similarity between transcripts *i* and *j*, was introduced into the model by incorporating a nondiagonal transcript covariance matrix ${{\varvec{V}}}_{M1}$. The covariance between transcripts was expressed by the Jaccard similarity coefficient: $J\left(A,B\right)=\frac{\left|A\cap B\right|}{\left|A\cup B\right|},$ which in the case of transcripts was calculated based on the similarity in their exon composition:

$$J\left(i,j\right)=\frac{a}{N},$$

where *a* represents the number of exons common between transcripts *i* and *j*, while *N* represents the total number of exons of a given gene. Independence was assumed between genes, so the resulting matrix had a block diagonal structure. Transcript information was obtained from the Ensembl database, release100^21^ and Jaccard coefficients were calculated using the ADE-4 package^22^.

The *gene-based model* (M2) was applied to the same dependent variable (${\varvec{y}}$) as M1 and is given by:

2 $${\varvec{y}}={\varvec{\mu}}+{{\varvec{Z}}}_{M2}{\varvec{g}}+{{\varvec{e}}}_{M2},$$

where ${\varvec{g}}$ is the random gene effect with a preimposed normal distribution defined by $N\left(0,{{{\varvec{V}}}_{M2}\sigma }_{g}^{2}\right)$, ${{\varvec{e}}}_{M2}$ is a vector of random residuals distributed as $N\left(0,{{\varvec{I}}\sigma }_{{e}_{M2}}^{2}\right)$, ${{\varvec{Z}}}_{M2}$ is an incidence matrix for ${\varvec{g}}$. No covariance between genes was assumed so ${{\varvec{V}}}_{M2}$ was diagonal.

Furthermore, the *Gene Ontology-based model* (M3):

3 $${\varvec{y}}={\varvec{\mu}}+{{\varvec{Z}}}_{M3}{\varvec{g}}{\varvec{o}}+{{\varvec{e}}}_{M3},$$

where ${\varvec{g}}{\varvec{o}}$ is the random effect of Gene Ontology (GO; geneontology.org) terms assigned to transcripts whose log_2_FC of transcript expressions are contained in ${\varvec{y}}$. It was assumed that ${\varvec{g}}{\varvec{o}}$ follows the normal distribution $N\left(0,{{{\varvec{V}}}_{M3}\sigma }_{go}^{2}\right)$, ${{\varvec{e}}}_{M3}$ is a vector of residuals distributed as $N\left(0,{{\varvec{I}}\sigma }_{{e}_{M3}}^{2}\right)$, and ${{\varvec{Z}}}_{M3}$ is an incidence matrix for the ${\varvec{g}}{\varvec{o}}$ terms. ${{\varvec{V}}}_{M3}$ describes the covariance between GO terms expressed, as above, by the Jaccard coefficients quantifying genes overlapping between two given GO terms. Each transcript was assigned GO term(s) from the biological process ontology, considering the ontologies from the 2nd hierarchy level.

Finally, a *model incorporating the effects of Reactome pathways* (M4) was fitted to transcript log_2_FC:

4 $${\varvec{y}}={\varvec{\mu}}+{{\varvec{Z}}}_{M4}{\varvec{r}}+{{\varvec{e}}}_{M4},$$

in this model, ${\varvec{r}}$ represents the random effect of Reactome pathways (reactome.org) corresponding to transcripts. The normal distribution pre-imposed on ${\varvec{r}}$ is given by $N\left(0,{{{\varvec{V}}}_{M4}\sigma }_{r}^{2}\right)$, ${{\varvec{e}}}_{M4}$ is a vector of residuals distributed as $N\left(0,{{\varvec{I}}\sigma }_{{e}_{M4}}^{2}\right)$, and ${{\varvec{Z}}}_{M4}$ is an incidence matrix assigning Reactome pathways to transcripts. The covariance matrix between Reactome pathways (${{\varvec{V}}}_{M4}$) was also expressed by the Jaccard coefficients quantifying genes overlapping between the two given pathways.

### Estimation of variance components and significance testing

The expectation–maximization algorithm^23^ was applied for the estimation of variance components underlying the four above models (i.e. ${\sigma }_{t}^{2},{\sigma }_{g}^{2},{\sigma }_{go}^{2},{\sigma }_{r}^{2},{\sigma }_{{e}_{M1}}^{2},{\sigma }_{{e}_{M2}}^{2},{\sigma }_{{e}_{M3}}^{2}$ and ${\sigma }_{{e}_{M4}}^{2}$) and the mixed model equations^24^ were used to obtain solutions of ${\varvec{\mu}},{\varvec{t}},{\varvec{g}},{\varvec{g}}{\varvec{o}}$ and ${\varvec{r}}$:

$$\left[\begin{array}{c}\widehat{{\varvec{\mu}}}\\ \widehat{{\varvec{x}}}\end{array}\right]={\left[\begin{array}{cc}{\varvec 1}^{T}{{\varvec{R}}}^{ -1}\varvec1& {\varvec 1}^{T}{{\varvec{R}}}^{-1}{{\varvec{Z}}}_{x}\\ {{\varvec{Z}}}_{x}^{T}{{\varvec{R}}}^{-1}\varvec 1& {{\varvec{Z}}}_{x}^{T}{{\varvec{R}}}^{-1}{{\varvec{Z}}}_{x}+{{\varvec{G}}}^{-1}\end{array}\right]}^{-1}\left[\begin{array}{c}{\varvec 1}^{T}{{\varvec{R}}}^{-1}{\varvec{y}}\\ {{\varvec{Z}}}_{x}^{T}{{\varvec{R}}}^{-1}{\varvec{y}}\end{array}\right],\mathrm{ where }\,{\varvec{R}}={\varvec{I}}{\widehat{\sigma }}_{{e}_{Mi}}^{2}\mathrm{ and }{\varvec{G}}={{\varvec{V}}}_{{\varvec{x}}}{\widehat{\sigma }}_{x}^{2},$$

with ${\varvec{x}}$ representing ${\varvec{t}},{\varvec{g}},{\varvec{g}}{\varvec{o}}$ or ${\varvec{r}}$ depending on the model, and $i\in \left\{M1, M2, M3, M4\right\}$ being a model indicator. Each element of the solution vectors $\widehat{{\varvec{t}}},\widehat{{\varvec{g}}},\widehat{{\varvec{g}}{\varvec{o}}}$ and $\widehat{{\varvec{r}}}$ was transformed to the standard normal distribution and tested for significance by assessing the probability of obtaining a more extreme value based on the standard normal density function.

### Enhancement of the computational efficiency of the estimation

In order to maximise the computational performance of the estimation of model parameters and its variance components, a custom-written Python program implementing the Numba library^25^ was used. Numba compiles a subset of native Python and NumPy code into the machine code. Since all calculations were carried out on a multicore server, the Numba library was also used to parallelize the code, what further improved the computing time compared to a native Python application.

### Ethics approval

Not applicable, only in silico data was processed in this study.

## Results

Obviously models M1 and M2, fitting thousands of components, i.e. transcripts (M1) or genes (M2) explain a much larger proportion of the observed variability of log_2_FC than the M3 and M4 fitting the functional components, i.e. GO terms (M3) or Reactome pathways (M4). Still, by taking into account the correlation between transcripts expressed by their exon composition, we explain 12.43% and 18.32% of the total variability of log_2_FC observed in liver and adrenal tissues, respectively. A similar picture arises when the assumed source of variability of log_2_FC is considered on a gene level, resulting in 10.12% and 16.45% variance components in liver and adrenal tissues respectively—somewhat lower than for correlated transcripts in M1. By further shrinking the functional units to GO terms and Reactome pathway units we explain “only” 1.48% (GO) and 1.45% (Reactome) of the total variance for liver, as well as 1.44% (GO) and 1.93% (Reactome) for the adrenal tissue (Table 1).

**Table 1:** Variance components estimated by the mixed models (M1–M4) expressed as the percentage of the total variance of ***y***.

Tissue	$\frac{{\sigma }_{t}^{2}}{{\sigma }_{y}^{2}}$ (M1) (%)	$\frac{{\sigma }_{g}^{2}}{{\sigma }_{y}^{2}}$ (M2) (%)	$\frac{{\sigma }_{go}^{2}}{{\sigma }_{y}^{2}}$ (M3) (%)	$\frac{{\sigma }_{r}^{2}}{{\sigma }_{y}^{2}}$ (M4) (%)
Liver	12.43	10.12	1.48	1.45
Adrenal	18.32	16.45	1.44	1.93

${\sigma }_{t}^{2}$ the transcript variance, ${\sigma }_{g}^{2}$ the gene variance, ${\sigma }_{go}^{2}$ the GO term variance, ${\sigma }_{r}^{2}$ the Reactome pathway variance, ${\sigma }_{y}^{2}$ the variance of log_2_FC.

Considering the transcript and gene levels, in the liver, M1 and M2 point at the significant effects of the *PNKD* and *TRIP12* genes. *PNKD* exhibits a significant effect of a transcript ENSRNOT00000046229, which is approximately 2^23^ times higher expressed in the control than in the heat-stress group. However, three of the five transcripts of the gene show higher expression in the heat-stress group, reaching 2^5^ higher expression of ENSRNOT00000089580. *TRIP12* synthesises one particular transcript (ENSRNOT00000022822) which is 2^22^ times higher expressed in the control than in the heat-stress group. Different genes were significantly differentially expressed in the adrenal tissue. One transcript of the *SUCO* gene (ENSRNOG00000026542) is 2^23^ times higher expressed in the control than in the heat-stress group, while the three other transcripts synthesised from this gene have an opposite effect, i.e. show higher expression in heat-stressed individuals. *PLEC* has two transcripts which are significantly overexpressed in the heat-stress group. *PER3* was significant only on the gene level (M2) with all of its transcripts being under expressed during heat stress (Table 2).

**Table 2:** Top 5 significant transcripts from M1, top 2 significant genes from M2, significant Gene Ontology terms from M3, and significant Reactome pathways from M4.

Tissue	Model	ID name	Effect	P
Liver	Transcript (M1)	ENSRNOT00000074131 *PNKD*	2.76	4.7 × 10^–10^
	Transcript (M1)	ENSRNOT00000093245 *TRIP12*	2.15	1.2 × 10^–6^
	Transcript (M1)	ENSRNOT00000093735 *TRIP12*	1.67	0.00016
	Transcript (M1)	ENSRNOT00000022822 *TRIP12*	1.65	0.00021
	Transcript (M1)	ENSRNOT00000079452 *TRIP12*	1.63	0.00023
	Gene (M2)	ENSRNOG00000016963 *TRIP12*	1.66	3.5 × 10^–5^
	Gene (M2)	ENSRNOG00000014806 *PNKD*	1.12	0.00520
	Gene ontology (M3)	GO:1901315 Negative regulation of histone H2A K63-linked ubiquitination	0.52	0.00055
	Gene ontology (M3)	GO:2000780 Negative regulation of double-strand break repair	0.48	0.00130
	Gene ontology (M3)	GO:2000779 Regulation of double-strand break repair	0.37	0.01300
	Gene ontology (M3)	GO:0045995 Regulation of embryonic development	0.30	0.04200
	Gene ontology (M3)	GO:0002181 Cytoplasmic translation	0.29	0.05200
	Reactome pathway (M4)	R-RNO-6791226 Major pathway of rRNA processing in the nucleolus and cytosol	0.29	0.05500
Adrenal	Transcript (M1)	ENSRNOT00000075998 *SUCO*	3.48	9.2 × 10^–11^
	Transcript (M1)	ENSRNOT00000084058 *SUCO*	2.18	4.9 × 10^–5^
	Transcript (M1)	ENSRNOT00000082271 *PLEC*	2.10	8.9 × 10^–10^
	Transcript (M1)	ENSRNOT00000075936 *SUCO*	1.95	0.00029
	Transcript (M1)	ENSRNOT00000088945 *PLEC*	1.70	0.00160
	Gene (M2)	ENSRNOG00000026542 *SUCO*	2.26	8.4 × 10^–6^
	Gene (M2)	ENSRNOG00000018413 *PER3*	1.63	0.00130
	Reactome pathway (M4)	R-RNO-212436 Generic Transcription Pathway	0.49	0.00500

*ID* the Ensembl transcript ID, *Ensembl gene ID* Gene Ontology database ID, or Reactome ID, depending on the effect considered, *Effect* represents the estimate from the corresponding mixed model M1–M4, *P* P value corresponding to the normal probability density function with mean zero and the variance estimated by the corresponding model.

While moving to a functional level, we did not identify any significant GO terms differentially expressed in the adrenal tissue and a single Generic Transcription Reactome Pathway (R-RNO-212436). However, in the liver, five ontologies and one Reactome pathway were significant. Functionally, they can be grouped into ontologies related to DNA repair through the regulation of double-strand break repair (GO:2000780 and GO:2000779) and histone ubiquitination (GO:1901315), to the regulation of embryonic development (GO:0045995) and to cytoplasmic translation (GO:0002181, R-RNO-6791226).

## Discussion

Various scopes of information considered in our study explain different amounts of the observed variation of transcript differential expressions across the genome (Table 1). The difference in the estimated variance components related to transcripts, genes, GO terms and Reactome pathways associates with the number of levels of those independent variables. Since we are not aware of the corresponding estimates reported by other studies, a discussion with the literature is not possible. Therefore, at this stage, we can only hypothesise that on the one hand, the differences in variance components can be due to the technical nature of the model, since many more transcript effects were estimated as Reactome pathway effects. On the other hand, those effects were modelled as random and thus their variation was constrained by the predefined co-variance structure so that the differences in variance components express the biological phenomenon—Reactome pathways aggregate effects of many transcript/genes.

By considering the significant transcripts, genes and pathways identified in our study, a consistent picture emerges that DNA stability and its repair mechanisms are affected by heat stress. This phenomenon has already been reported by other authors, e.g.^26^ and was reviewed by Kantidze et al.^27^. On a gene level, *TRIP12* was reported as encoding a protein which is associated with chromatin and plays a role in the maintenance of genome integrity^28^ and *PER3* influences DNA damage repair by correlating with checkpoint kinase 2 gene^29^. Mutations in the *SUCO* gene induce hypoglycaemia [www.ensembl.org] in humans, which is the cause of hypothermia in diabetic patients^30^. *PER3* was differentially expressed in broiler chicken maintained under control and heat stress conditions^31^. In general, it has been proven that *PER3* is associated with behavioural differences towards stress and was influenced by stress and ethanol treatment in BXD strain mice^32^.

On the functional level, we observed a significantly under expression of genes with ontologies related to double-strand break repair (GO:2000780 and GO:2000779) and histone ubiquitination. Furthermore, outside of the nucleus, a significant effect of the cytoplasmic translation ontology (GO:0002181) and rRNA processing in the nucleolus and cytosol pathway (R-RNO-6791226) can be linked to the phenomenon of the aggregation of proteins in the cytoplasm of yeast cells subjected to heat stress, which consequence was impaired cytoplasmic translation^33^.

Noteworthy, our list of significant transcripts, genes, GO terms, and Reactome pathways did not overlap with the results reported for the same material by Dou et al.^12^. Only the *PER3* gene was significantly associated with differential expression in adrenal tissue in both studies. The most emerging difference concerned the number of significant effects reported. While Dou et al.^12^ estimated 3909 and 4953 significantly differentially expressed genes for liver and adrenal tissues respectively, our study pointed at only two significant genes for each tissue. Similarly, the number of significant GO terms was 193 for liver and 79 for adrenal tissue in Dou et al.^12^, while only five and zero in our results. The observed differences are caused by the following factors: different raw reads editing criteria, different approaches to estimate transcript expression, and different statistical modelling of expression data. Dou et al.^12^ used the Cufflinks software^34^ for bioinformatic processing of the expression data, which implements Tophat2^35^ for the alignment of reads to the reference genome and a single gene hypothesis test with multiple testing correction of P values via the FDR for the assessment of differential expression. In our analysis, an alignment-free approach and a random effect model incorporating all transcripts/genes simultaneously were applied. Already ^36^ pointed at differences in the statistical inference based on fixed and random effect models, indicating that the former tend to underestimate, and the latter—overestimate a residual variance. As a consequence, a type I-error is often elevated in fixed-effect models, which was analytically demonstrated by ^37^ in the context of meta-analysis. Moreover, significant differences in the quantification of expression between alignment-based and alignment-free approaches were recently demonstrated in our unpublished analysis of *Sus scrofa* RNA-seq data by Hoffman et al.

## Conclusions

Mixed models, i.e. statistical models fitting random effects, are a valuable tool for the analysis of high-throughput biological data. Their major advantages comprise: (1) the possibility to incorporate information on covariance between observations, which is often neglected while applying simple, fixed effect models, and (2) circumventing the problem of multiple testing, by simultaneous fitting all effects. We see the major limitation of the proposed approach in the varying quality of functional annotation of genomes available for different species. While genomes of humans and experimental species, such as rats, are very well functionally annotated with the most of transcripts/genes assigned to GO terms and metabolic pathways, less well-studied species have less complete annotation, which would enforce the incorporation of phantom GO and phantom pathway effects (similarly to phantom parent groups in livestock genetic evaluation).

From the biological perspective, *PER3* and *SUCO* genes as well as DNA repair and translation were indicated as factors playing a significant role in heat stress response.

## Data Availability

The datasets generated and/or analysed during the current study are not publicly available due to institutional constrains, but are available from the Yachun Wang on reasonable request.

## Code availability

Python code is available in principle upon request from the corresponding author.
